# Adaptive Time Propagation for Time-dependent Schrödinger equations

**DOI:** 10.1007/s40819-020-00937-9

**Published:** 2020-12-19

**Authors:** Winfried Auzinger, Harald Hofstätter, Othmar Koch, Michael Quell

**Affiliations:** 1 TU Wien, Institute of Analysis and Scientific Computing, Wiedner Hauptstraße 8–10, 1040 Vienna, Austria; 2 University of Vienna, Institute of Mathematics, Oskar-Morgenstern-Platz 1, 1090 Vienna, Austria; 3 TU Wien, Institute for Microelectronics, Gußhausstraße 27–29, 1040 Vienna, Austria

**Keywords:** Time-dependent Schrödinger equations, Splitting methods, Magnus-type integrators, Adaptive stepsize selection

## Abstract

We compare adaptive time integrators for the numerical solution of linear Schrödinger equations where the Hamiltonian explicitly depends on time. The approximation methods considered are splitting methods, where the time variable is split off and advanced separately, and commutator-free Magnus-type methods. The time-steps are chosen adaptively based on asymptotically correct estimators of the local error in both cases. It is found that splitting methods are more efficient when the Hamiltonian naturally suggests a separation into kinetic and potential part, whereas Magnus-type integrators excel when the structure of the problem only allows to advance the time variable separately.

## Introduction

We study systems of linear ordinary differential equations of Schrödinger type

1 $$\begin{aligned} {\left\{ \begin{array}{ll} \psi '(t) = -\,{\mathrm {i}}\,H(t)\,\psi (t)\,, \qquad t \in [t_0, t_{{\mathrm {end}}}]\,, \\ \psi (t_0) = \psi _0\; \text { given}\,, \end{array}\right. } \end{aligned}$$

with a time-dependent Hermitian matrix $H :{\mathbb {R}} \rightarrow {\mathbb {C}}^{d \times d}$. The exact flow of (1) is denoted by ${\mathcal {E}}(t,u_0)$ in the following. High-dimensional systems of this form arise for instance in the design of oxide solar cells [31], describing the movement and interaction of electrons within Hubbard-type models of solid state physics, where the explicit time-dependence here originates from an external electric field associated with the impact of a photon, or as typical semiclassical models arising in quantum control [40]. In the former application, the computational challenge results from the high dimension of the resulting system. Indeed, for a model with *n* discrete locations, the state space has dimension $4^n$. Thus, for a model with the claim of physical relevance, the problem quickly reaches the limitations of modern supercomputers. In the latter application, the semiclassical parameter is chosen as very small, which mandates a fine spacial discretization, again resulting in very large systems of ODEs. Thus the main motivation for the present study is to identify the computationally most efficient numerical time integrators for the considered problem class in order to make large-scale simulations of high accuracy feasible on current computer hardware.

The aim of this paper is to compare two approaches to the numerical time integration of problems of the form (1). Popular integrators for time-dependent linear homogeneous differential equations are based on the Magnus expansion [23, 35], or on commutator-free exponential-based integrators [1]. These have been found to excel over classical Magnus integrators (introduced as numerical methods in [21]) for example in [3] and will therefore be used in the present study. In contrast, non-autonomous problems can also be solved by interpreting the independent variable *t* as a separate component, which in splitting methods can be frozen over a time-step and propagated separately. This approach is discussed extensively in [16–18, 20, 39] and references therein. The success of the splitting approach critically depends on the structure of the underlying problem. If the operator *H*(*t*) naturally suggests a splitting, where the time-dependent part is cheap to compute for fixed *t*, this may offer computational advantages when *t* is propagated along with one sub-operator. However, if only *t* is split off, the required number of compositions in a splitting approach may be prohibitive from a computational point of view. Also, if *H* has a special structure which can be exploited to increase the efficiency, the introduction of the additional variable *t* may destroy this structure [16]. We will corroborate these general observations on a number of practical examples, see also [19] for an abstract discussion of the computational effort.

The present comparisons involve methods that have been used in previous studies, but not in comprehensive assessment of the efficiency when applied to a number of application-motivated examples. The efficiency of adaptive splitting methods has been studied by the authors for instance in [2, 9], and adaptive Magnus-type methods are discussed in [3, 7]. This work adds the aspect of understanding *adaptive* splitting and Magnus-type methods as to their applicability and respective merits. By providing a meticulous comparison on several significant examples from applications, we give a balanced account of advantages and disadvantages of the two numerical approaches. The Hubbard model is of high interest in solid state physics, and therefore a search for the best numerical approach among several contenders is of relevance.

In Sect. 2 of this manuscript, we specify the model model problems that we will subsequently resort to in our comparisons, in Sect. 3 we briefly recapitulate the numerical approaches that are used, and in Sect. 4 we give the results of our numerical comparisons. The main criterion to assess the computational efficiency is the required CPU time to reach a prescribed accuracy, as the considered numerical approaches are fundamentally different in their structure and do not readily admit other metrics.

## Model Problems

We consider a Rosen–Zener model related to quantum optics, a Hubbard model of the impact of light on a solid, and a semiclassical problem typical for quantum control.

*Rosen–Zener problem* As the first example, we consider a Rosen–Zener model from [19], which appears in quantum optics, see also [33]. The associated Schrödinger equation in the interaction picture is given by (1) with

2a $$\begin{aligned}&H(t) = f_1(t) \sigma _1 \otimes I_{k\times k} + f_2(t) \sigma _2 \otimes R \in {\mathbb {C}}^{2k\times 2k},\quad k=50\,, \end{aligned}$$

2b $$\begin{aligned}&\sigma _1 = \left( \begin{array}{cc} 0 &{} 1 \\ 1 &{} 0 \end{array} \right) ,\qquad \sigma _2 = \left( \begin{array}{cc} 0 &{} -{\mathrm {i}}\\ {\mathrm {i}}&{} 0 \end{array} \right) ,\end{aligned}$$

2c $$\begin{aligned}&R = {\mathrm {tridiag}}(1,0,1) \in {\mathbb {R}}^{k\times k},\end{aligned}$$

2d $$\begin{aligned}&f_1(t) = V_0 \cos (\omega t) \left( \cosh (t/T_0) \right) ^{-1},\quad f_2(t) = V_0 \sin (\omega t) \left( \cosh (t/T_0) \right) ^{-1},\end{aligned}$$

2e $$\begin{aligned}&\omega =\tfrac{1}{2},\ T_0 =1,\ V_0 = 1, \end{aligned}$$

where the initial condition is chosen as $\psi (0)=(1,\dots ,1)^T.$ The integration interval is $t \in [-5,5]$.

*Hubbard model for solar cells* Next, we consider a Hubbard model describing the movement and interaction of electrons within an oxide solar cell [25, 31] built from ${{\mathrm {LaVO}}}_3$, with^1^$H(t)\in {\mathbb {C}}^{4900\times 4900}$. The explicit time-dependence here originates from an external electric field associated with the impact of a photon.

This model is given by a finite-dimensional Hamiltonian in second quantization of the form

3 $$\begin{aligned} H=\frac{1}{2} \sum _{ij \sigma } v_{ij} c_{j \sigma }^\dagger c_{i \sigma }^{} + \sum _{ij \sigma \sigma '} U_{ij} {{\hat{n}}}_{i\sigma } {{\hat{n}}}_{j\sigma '}. \end{aligned}$$

Here, the annihilation and creation operators $c_{i \sigma }^{}$ and $c_{j \sigma }^\dagger$ take an electron away from site *i* with spin $\sigma \in \{\uparrow ,\downarrow \}$ and add it on site *j*.

The impact of a photon exciting the system out of equilibrium can be described by a classical electric field pulse, which introduces time-dependence to the Hamiltonian (3), see [25]. We choose ${\mathrm {e}}^{{\mathrm {i}} \,\omega (t)}$ with $\omega (t) =\tfrac{1}{10} \exp \left( -\tfrac{1}{6} (t-6)^2\cos \left( \tfrac{7\pi }{4}(t-6)\right) \right)$, which appears in off-diagonal entries of *H*(*t*) depending on the geometry underlying the model of the investigated solid. The model is described in detail in [27].

The oscillating and quickly attenuating electric field generated by the external potential in this model makes adaptive time-stepping a relevant issue. Time integration proceeds on the interval $t\in [0,30]$.

*Quantum control* A model typical for quantum control of atomic systems which is discussed in [28], see also [40], introduces a potential which explicitly depends on time,

4a $$\begin{aligned}&{\mathrm {i}}\,\partial _t \psi (x,t) = \varepsilon {\varDelta }\psi (x,t) + \varepsilon ^{-1} V(x,t)\,\psi (x,t)=H\psi (x,t)\,, \qquad t>0\,, \end{aligned}$$

4b $$\begin{aligned}&\psi (x,0) = \psi _0(x)\,, \end{aligned}$$

with *V*(*x*, *t*) and the initial condition chosen as

$$\begin{aligned} \psi _0(x)= & {} (\delta \pi )^{-\frac{1}{4}}{\mathrm {e}}^{\frac{{\mathrm {i}}\,k_0(x-x_0)}{\delta }-\frac{(x-x_0)^2}{2\delta }},\\ V(x,t)= & {} V_0(x)+\rho (3t-1)\rho (\sin (2\pi (x-t))) + 10^{-3} t \sin (20\pi x),\\ V_0(x)= & {} \rho (4x)\sin (20\pi x),\\ \rho (x)= & {} {\left\{ \begin{array}{ll} e^{\frac{-1}{1-x^2}}, &{} |x|<1\\ 0, &{}\text {otherwise,} \end{array}\right. } \end{aligned}$$

where $x_0=-0.3$, $k_0=0.1$, $\delta =10^{-3}$ and $\varepsilon$ in (4a) assumes the values $2^{-6},\ 2^{-8},\ 2^{-10},\ 2^{-12}$. The spatial interval $[-1,1]$ is discretized using a Fourier pseudospectral method at 2048 points for periodic boundary conditions. The computation terminates at $t_{{\mathrm {end}}}=0.75.$

## Adaptive Time Integration

### Splitting Methods

Splitting methods constitute a popular *divide-and-conquer* approach for numerical time integration of (1) when the Hamiltonian is partitioned, i.e., $-{\mathrm {i}}H(t) = A(t) + B(t)$ and the operators *A* and *B* have different properties which promise computational advantages when propagated independently. This is for instance typical for the splitting of a Schrödinger operator into kinetic and potential part.

In our context, we will use splitting methods by making the problem formally autonomous by considering *t* as an additional solution component and adding the equation $t'=1$. In this setting, time can be advanced separately, or simultaneously with one suboperator if this is autonomous. More precisely, in the definition of the splitting, the operators become

$$ \psi \longleftarrow \left( \begin{array}{c} \psi \\ t \end{array} \right) ,\quad A \longleftarrow \left( \begin{array}{c} A \\ 1 \end{array} \right) ,\quad B \longleftarrow \left( \begin{array}{c} B \\ 0 \end{array} \right) . $$

The same holds *mutatis mutandis* when *t* is propagated together with *B*.

For autonomous problems, splitting methods have the following form: At the (time-)semi-discrete level, *s*-stage exponential splitting methods use multiplicative combinations of the partial flows ${\mathcal {E}}_A(t,u_0):u_0 \mapsto u(t)$ with $u'(t)=A(u(t)),\ u(t_0)=u_0$, and ${\mathcal {E}}_B(t,u_0):u_0 \mapsto u(t)$ with $u'(t)=Bu(t),\ u(t_0)=u_0$. For a single step $(t_0,u_0) \mapsto (t_0+h,u_1)$ with time-step $t=h$, this reads

5 $$\begin{aligned} u_{1} := {\mathcal {S}}(h,u_0) = {\mathcal {E}}_B(b_s h,\cdot ) \circ {\mathcal {E}}_A(a_s h,\cdot ) \circ \ldots \circ {\mathcal {E}}_B(b_1 h,\cdot ) \circ {\mathcal {E}}_A(a_1 h,u_0), \end{aligned}$$

where the coefficients $a_j,b_j,\, j=1 \ldots s$ are determined from *order conditions* to achieve a desired order of consistency [23].

### Local Error Estimators for Splitting Methods

As the basis for adaptation of the time-steps, three classes of local error estimators are used in this study. These have different advantages depending on the context in which they are applied [5].

(i) *Embedded pairs of splitting formulae* have first been considered in [32] and are based on reusing a number of evaluations from the basic integrator. In this paper, we will focus on the pairs [6, Emb 4/3 AK p] of orders four and three and [6, Emb 5/4 AK (ii)] of orders five and four, which were found to be the most successful in earlier work [2].
(ii) A *defect-based* error estimator has been proposed and analyzed in [8, 10–12]. In order to construct an error estimator associated with a splitting method of order $p \ge 1$, an integral representation of the local error involving the defect ${\mathcal {D}}$ of the numerical approximation is evaluated by means of an Hermite quadrature formula. Due to the fact that the validity of the *p*-th order conditions ensures that the first $p-1$ derivatives of ${\mathcal {D}}$ vanish at $t=t_0$, this leads to a local error estimator involving a single evaluation of the defect,
  6 $$\begin{aligned} {\mathcal {P}}(t,u_0) = \tfrac{1}{p+1}\,t\,{\mathcal {D}}(t,u_0) \,\approx \, {\mathcal {L}}(t,u_0) = {\mathcal {S}}(t,u_0)-{\mathcal {E}}(t,u_0)\,. \end{aligned}$$
  This device works generally for splittings of any order into an arbitrary number of operators if Fréchet derivatives of the subflows are available, see [4]. We use the defect-based error estimator in conjunction with the integrators in [6, Emb 4/3 AK p] and [6, Emb 5/4 AK (ii)], since these are close to optimal.
(iii) For *adjoint pairs of formulae* of odd order *p*, an asymptotically correct error estimator can be computed at the same cost as for the basic method, see [5]. Since the error estimator is easy to construct and evaluate in this case, we employ the pair [6, PP 5/6 A] of orders 5/6, since this was found to be efficient for high accuracy demands for instance in [2]. We will also employ this optimized method in conjunction with the defect-based error estimator for reasons of comparison.

All the error estimates we use in our comparisons are *asymptotically correct*, i.e., the deviation of the error estimator from the true error tends to zero faster than does the error.

### Commutator-free Magnus-type Integrators

A successful and much used class of integration methods is given by higher-order commutator-free Magnus-type integrators (CFM) [1, 22]. These approximate the exact flow in terms of products of exponentials of linear combinations of the system matrix evaluated at different times, avoiding evaluation and storage of commutators. These have been found to excel over classical Magnus integrators in applications in our interest in [3].

One step of a CFM scheme for (1) starting at $(t_0,u_0)$ is defined by^2^

$$\begin{aligned} u_{1} = {\mathcal {S}}(\tau ;t_0)\,u_0\,, \end{aligned}$$

with the ansatz [1, 22]

7 $$\begin{aligned} \begin{aligned} {\mathcal {S}}(\tau ;t_0)&= {\mathcal {S}}_J(\tau ) \cdots {\mathcal {S}}_1(\tau ) = {\mathrm {e}}^{{\varOmega }_J (\tau )} \,\cdots \, {\mathrm {e}}^{{\varOmega }_1(\tau )}\,, \\[1mm] {\varOmega }_j(\tau )&= \tau B_j(\tau ),~~ j=1, \ldots , J, \\ B_{j}(\tau )&= \sum _{k=1}^K a_{jk}\, H_{k}(\tau ),\quad H_{k}(\tau ) = -{\mathrm {i}}H(t_0+c_k \tau )\,, \end{aligned} \end{aligned}$$

where the coefficients $a_{jk}$, $c_k$ are determined from the *order conditions* (a system of polynomial equations in the coefficients) such that the method attains convergence order *p*, see for example [19] and references therein. Algorithms to efficiently generate the order conditions are described for instance in [26]. Since such a system of equations generally does not define a unique solution, numerical optimization techniques are employed, for example minimizing the *leading local error term* of the resulting integrator.

In this study, we will use the methods referred to as CF4oH and CF6n in [3].

The choice of the methods above for our comparisons is motivated by the fact that these two methods were found to be the most efficient CFM methods in the study [3].

### Local Error Estimation for Magnus-type Methods

As a basis for adaptive time-stepping, defect-based error estimators for CFM methods and for classical Magnus integrators have been introduced in [7]. For the defect

8 $$\begin{aligned} {\mathcal {D}}(\tau )={\mathcal {S}}'(\tau ;t_0)-A(t_0+\tau ){\mathcal {S}}(\tau ;t_0) \end{aligned}$$

it holds that

$$\begin{aligned} {\mathcal {D}}(0)={\mathcal {D}}'(0)=\cdots ={\mathcal {D}}^{(p-1)}(0)=0, \end{aligned}$$

for an order *p* method.

The local error ${\mathcal {L}}(\tau )\psi _0:=({\mathcal {S}}(\tau ;t_0)-{\mathcal {E}}(\tau ;t_0))\psi _0$ can be expressed via the *variation-of-constant formula* as

$$\begin{aligned} {\mathcal {L}}(\tau )=\int _0^\tau {\varPi }(\tau ,\sigma ){\mathcal {D}}(\sigma )\,{\mathrm {d}}\sigma ={{\mathscr {O}}}(\tau ^{p+1}),\quad {\varPi }(\tau ,\sigma )={\mathcal {E}}(\tau -\sigma ;t_0+\sigma ). \end{aligned}$$

For the practical evaluation of the defect, the derivative of matrix exponentials of the form

$$ \tfrac{{\mathrm {d}}}{{\mathrm {d}}\tau }{\mathrm {e}}^{\tau B(\tau )} = {\varGamma }(\tau )\,{\mathrm {e}}^{\tau B(\tau )} $$

is required. The function ${\varGamma }$ can be expressed as an infinite series or alternatively as an integral. These are approximated by truncation or numerical Hermite quadrature, respectively, to yield a computable quantity ${{\tilde{{\varGamma }}}}$ and an approximate defect ${\tilde{{\mathcal {D}}}}$. The resulting computable error estimator is denoted by ${\tilde{{\mathcal {P}}}}$. The *asymptotical correctness* of the error estimators was established in [7].

In the numerical experiments reported in Sect. 4 below, truncation of the Taylor expansion has been used throughout.

### Adaptive Lanczos Method

The crucial computational step in any of the Magnus-type methods described above, is the evaluation of the action of a matrix exponential

9 $$\begin{aligned} E(t)v={\mathrm {e}}^{-{\mathrm {i}}t {\varOmega }}v,\quad {\varOmega }\, \text {Hermitian}, \, t \, \text {fixed}. \end{aligned}$$

Note that this subproblem is also solved in the splitting approximation of the problems (2) and (3), whereas in (4), the pseudospectral space discretization renders this substep the trivial exponentiation of a diagonal matrix. The standard Krylov approximation to ${\mathrm {e}}^{-{\mathrm {i}}t {\varOmega }} v$ reads

10 $$\begin{aligned} S_m(t)v = V_m\,{\mathrm {e}}^{-{\mathrm {i}}t T_m}\,V_m^*\,v = V_m\,{\mathrm {e}}^{-{\mathrm {i}}t T_m}e_1, \end{aligned}$$

with $T_m=(\tau _{i,j})$ tridiagonal and $V_m$ an orthonormal basis of the Krylov space ${\mathcal {K}}_m({\varOmega },v) = {\mathrm {span}}\{v,{\varOmega }v,\ldots ,{\varOmega }^{m-1}v\} \subseteq {\mathbf {C}}^n$. For Hermitian or skew-Hermitian matrices ${\varOmega }$, the Lanczos method [36] constitutes a computationally efficient realization.

In [30], a time-stepping strategy was introduced which is based on the defect of the approximation. Due to the success of this strategy documented ibidem, we use it invariantly in the Magnus-type integrators. The asymptotically correct error estimator is based on the *defect operator*

$$ D_m(t) = -{\mathrm {i}}\,{\varOmega }\,S_m(t) - S_m'(t) \in {\mathbf {C}}^{n \times n}. $$

The local error operator $L_m(t)=E(t)-S_m(t)$ can be represented as

$$ L_m(t)v = \int _0^t E(t-s)\,D_m(s)v\,{\mathrm {d}}s. $$

Numerical quadrature applied to this defect-based integral representation yields a computable, asymptotically correct local error bound satisfying (see [30]),

$$\begin{aligned}&\Vert L_m(t)v \Vert _2 \le \tau _{m+1,m}\gamma _m\,\frac{t^{m}}{m!},\\&\Vert L_m(t)v \Vert _2 = \tau _{m+1,m}\gamma _m\,\frac{t^m}{m!} + {{\mathcal {O}}}(t^{m+1}), \end{aligned}$$

with $$\gamma _m= \prod _{j=1}^{m-1} (T_m)_{j+1,j}.$$

As an error tolerance for the Lanczos matrix exponentiation, we prescribe $10^{-12}$. This allows to realize highly accurate time-stepping on the basis of this approximation with tolerance requirements as strict as $10^{-12}$.

### Step-size Selection

Based on a local error estimator, the time step-size is adapted such that the tolerance is expected to be satisfied in the following step. If $h_{\text {old}}$ denotes the present step-size, the next step-size $h_{\text {new}}$ in an order *p* method is predicted as (see [24, 37])

11 $$\begin{aligned} h_{\text {new}} = h_{\text {old}} \cdot \min \Big \{\alpha _{\text {max}},\max \Big \{\alpha _{\text {min}}, \alpha \,\Big (\dfrac{\text {tol}}{{\mathcal {P}}(h_{\text {old}})}\Big )^{\frac{1}{p+1}}\,\Big \}\Big \}, \end{aligned}$$

where we choose the parameters as $\alpha = 0.9$, $\alpha _{\text {min}} = 0.25$, $\alpha _{\text {max}} = 4.0$, and ${\mathcal {P}}(h_{\text {old}})$ is an asymptotically correct estimator for the local error arising in the previous time-step. This established and widely used strategy incorporates safety factors to avoid an oscillating and unstable behavior.

**Table 1:** Runtime for the Rosen–Zener model (2)

Scheme / tol=1.0e-5	#Steps	Time (s)
Emb 4/3 AK p	47	0.0216
Emb 4/3 AK p (defect 3)	47	0.0362
PP 5/6 A	23	0.0272
PP 5/6 A (defect)	23	0.0494
Emb 5/4 AK (ii)	23	0.0227
CF4oH	21	0.0144
CF6n	18	0.0270

Scheme / tol=1.0e-9	#Steps	Time (s)
Emb 4/3 AK p	451	0.1588
Emb 4/3 AK p (defect 3)	451	0.2707
PP 5/6 A	94	0.0715
PP 5/6 A (defect)	94	0.1501
Emb 5/4 AK (ii)	120	0.0719
CF4oH	106	0.0447
CF6n	55	0.0534

**Table 2:** Runtime for Hubbard model (3)

Scheme / tol=1.0e-5	#Steps	Time (s)
Emb 4/3 AK p	93	3.179
Emb 4/3 AK p (defect 3)	103	5.842
PP 5/6 A	61	4.125
PP 5/6 A (defect)	69	9.943
Emb 5/4 AK (ii)	63	4.527
CF4oH	57	2.600
CF6n	53	4.538

Scheme / tol=1.0e-9	#Steps	Time (s)
Emb 4/3 AK p	862	14.263
Emb 4/3 AK p (defect 3)	869	29.753
PP 5/6 A	273	11.725
PP 5/6 A (defect)	279	30.226
Emb 5/4 AK (ii)	372	12.424
CF4oH	237	7.831
CF6n	187	13.332

**Table 3:** Runtime for the quantum control problem (4) with $\varepsilon = 2^{-6}$

Scheme / tol=1.0e-5	#Steps	Time (s)
Emb 4/3 AK p	427	3.690
Emb 4/3 AK p (defect 3)	452	10.061
PP 5/6 A	257	4.553
PP 5/6 A (defect)	268	9.875
Emb 5/4 AK (ii)	89	1.074
CF4oH	97	38.048
CF6n	203	61.476

Scheme / tol=1.0e-9	#Steps	Time (s)
Emb 4/3 AK p	4282	36.712
Emb 4/3 AK p (defect 3)	4295	99.127
PP 5/6 A	1669	28.928
PP 5/6 A (defect)	1688	62.618
Emb 5/4 AK (ii)	561	6.709
CF4oH	450	38.890
CF6n	941	89.797

Scheme / tol=1.0e-12	#Steps	Time (s)
Emb 4/3 AK p	24082	200.399
Emb 4/3 AK p (defect 3)	24081	541.502
PP 5/6 A	5823	100.421
PP 5/6 A (defect)	5836	213.054
Emb 5/4 AK (ii)	2232	26.040
CF4oH	1495	66.152
CF6n	2973	180.930

**Table 4:** Runtime for the quantum control problem (4) with $\varepsilon = 2^{-8}$

Scheme / tol=1.0e-5	#Steps	Time (s)
Emb 4/3 AK p	282	2.443
Emb 4/3 AK p (defect 3)	287	6.508
PP 5/6 A	154	2.648
PP 5/6 A (defect)	155	5.642
Emb 5/4 AK (ii)	94	1.145
CF4oH	58	9.407
CF6n	62	15.277

Scheme / tol=1.0e-9	#Steps	Time (s)
Emb 4/3 AK p	2822	24.760
Emb 4/3 AK p (defect 3)	2822	65.917
PP 5/6 A	805	14.494
PP 5/6 A (defect)	807	30.469
Emb 5/4 AK (ii)	589	7.143
CF4oH	247	13.992
CF6n	254	23.956

Scheme / tol=1.0e-12	#Steps	Time (s)
Emb 4/3 AK p	15870	133.730
Emb 4/3 AK p (defect 3)	15867	359.906
PP 5/6 A	2636	44.502
PP 5/6 A (defect)	2637	96.960
Emb 5/4 AK (ii)	2347	27.547
CF4oH	780	28.341
CF6n	786	48.341

**Table 5:** Runtime for the quantum control problem (4) with $\varepsilon = 2^{-10}$

Scheme / tol=1.0e-5	#Steps	Time (s)
Emb 4/3 AK p	165	1.512
Emb 4/3 AK p (defect 3)	165	3.883
PP 5/6 A	79	1.408
PP 5/6 A (defect)	80	3.056
Emb 5/4 AK (ii)	60	0.773
CF4oH	43	3.425
CF6n	41	5.934

Scheme / tol=1.0e-9	#Steps	Time (s)
Emb 4/3 AK p	1642	13.641
Emb 4/3 AK p (defect 3)	1642	36.779
PP 5/6 A	389	7.084
PP 5/6 A (defect)	390	14.238
Emb 5/4 AK (ii)	374	4.329
CF4oH	162	6.320
CF6n	150	10.239

Scheme / tol=1.0e-12	#Steps	Time (s)
Emb 4/3 AK p	9238	76.601
Emb 4/3 AK p (defect 3)	9237	211.494
PP 5/6 A	1245	21.911
PP 5/6 A (defect)	1245	46.238
Emb 5/4 AK (ii)	1489	17.847
CF4oH	521	16.452
CF6n	403	22.347

**Table 6:** Runtime for the quantum control problem (4) with $\varepsilon = 2^{-12}$

Scheme / tol=1.0e-5	#Steps	Time (s)
Emb 4/3 AK p	181	1.565
Emb 4/3 AK p (defect 3)	181	4.173
PP 5/6 A	71	1.244
PP 5/6 A (defect)	72	2.694
Emb 5/4 AK (ii)	66	0.785
CF4oH	47	2.711
CF6n	53	4.678

Scheme / tol=1.0e-9	#Steps	Time (s)
Emb 4/3 AK p	1799	16.093
Emb 4/3 AK p (defect 3)	1799	42.375
PP 5/6 A	462	8.195
PP 5/6 A (defect)	559	20.996
Emb 5/4 AK (ii)	510	6.181
CF4oH	187	6.573
CF6n	183	10.729

Scheme / tol=1.0e-12	#Steps	Time (s)
Emb 4/3 AK p	10118	84.804
Emb 4/3 AK p (defect 3)	10116	227.637
PP 5/6 A	1485	24.911
PP 5/6 A (defect)	1607	58.523
Emb 5/4 AK (ii)	2116	24.399
CF4oH	653	20.688
CF6n	472	25.191

## Numerical Results

Here, we give the results of our experimental comparisons of the numerical methods described in Sect. 3. The numerical results have been obtained based on implementations which can be found at

https://github.com/HaraldHofstaetter/TimeDependentLinearODESystems.jl and https://github.com/HaraldHofstaetter/TSSM.jl.

As a measure of computational efficiency, we resort to CPU time on the *Vienna Scientific Cluster*. Its third generation cluster VSC-3 has 2020 nodes, each equipped with 2 processors (Intel Xeon E5-2650v2, 2.6 GHz, 8 cores). The runtimes we give below are averages over 100 identical runs on a single compute node, respectively. Runtime seems to be the most reasonable measure of computational efficiency due to the very different nature of the two numerical approaches. Two different local error tolerances $10^{-5}$ and $10^{-9}$ are prescribed for all examples, for (4) the tolerance $10^{-12}$ could also be reached.

*Rosen–Zener model.* In Table 1 we show the results for the Rosen–Zener model (2). For the splitting methods, only the time variable is split off and the Hamiltonian is exponentiated as a whole. In modern computer arithmetics, a conceivable splitting into real and imaginary part does not promise a computational advantage. We observe that the most efficient exponential-based method is CF4oH, while Emb 4/3 AK p is the best splitting method for the larger tolerance $10^{-5}$, and PP 5/6 A excels for tolerance $10^{-9}$. Note that the number of time-steps does not immediately correspond with the computational effort, the commutator-free Magnus-type method of order six requires the fewest steps, but is more expensive in each step and thus not the fastest integrator. The fastest exponential-based integrator is almost twice as fast as the best splitting method.

*Hubbard model.* For the Hubbard model of solar cells (3) we obtain a similar picture. Again, only the time variable is split off. Table 2 shows the runtimes for tolerances $10^{-5}$ and $10^{-9}$. The fourth order commutator-free Magnus-type integrator CF4oH is the most efficient for both tolerances, and again, Emb 4/3 AK p is the best splitting method for the larger tolerance, and PP 5/6 A for the stricter tolerance. The best exponential-based method again excels over the best splitting method.

*Quantum control.* The results for the semiclassical problem (4) show a different picture than the previous investigations. The reason is obvious: The problem (4) suggests a natural splitting into kinetic and potential part, and hence *t* can be propagated efficiently alongside with the autonomous kinetic operator. We vary $\varepsilon$ from $\varepsilon =2^{-6}$ to $\varepsilon =2^{-12}$ in Tables 3, 4, 5 and  6. For this example, a tolerance of $10^{-12}$ could additionally be achieved and is added to the numerical results. Throughout, the best splitting method is EMB 5/4 AK (ii), and the best Magnus-type method is CF4oH. For larger $\varepsilon$, splitting methods are clearly to be preferred, but this advantage is significantly diminished for the more oscillatory problems for smaller $\varepsilon$. Indeed, Magnus-type integrators are known to excel for oscillatory problems. For larger $\varepsilon$ and particularly larger tolerances, Emb 5/4 AK (ii) is by far more efficient than the best exponential-based method, but for smaller $\varepsilon$, this advantage is dimished, and for $\varepsilon =2^{-10}$ and $2^{-12}$ and tolerance $10^{-12}$, CF4oH is even slightly faster. The reason may be the additional splitting error which contributes to dimished efficiency due to reduced accuracy for a given computational effort.

## Conclusions

We have studied the differences between two fundamentally diverse approaches for the solution of linear non-autonomous systems of differential equations. Exponential-based methods related to the Magnus expansion are contrasted with splitting methods, where the time variable is split off and suitably propagated. Both approaches allow to construct asymptotically correct estimators for the local time-stepping error and implement adaptive time-stepping on this basis. Which method is more efficient depends on the problem structure. If only the time variable is split off, the additional substeps induced in the splitting procedure seem not to be justified from the point of view of computational efficiency. However, if the problem naturally suggests a splitting into a time-dependent and a time-independent part, the approach may be more efficient. However, for highly oscillatory problems, the splitting error is too large and Magnus-type integrators are again to be preferred. Our findings may also have an impact on the study of time-dependent differential equations of other classes such as differential-algebraic equations [15], functional and stochastic differential equations [34], or fractional differential equations [29, 38], see also [13, 14].

## Footnotes

1 The dimension of the matrix in this model grows exponentially with the number of considered sites in the Hubbard model of the solid, making the issue of an efficient time integrator crucial. For our illustrations in this paper, we choose a model of manageable size.

2 Note the slight difference in notation as compared to splitting methods, which is motivated by the fact that for time-dependent linear problems, the evolution depends on the initial time and represents a linear operator applied to the initial value.

## References

[1] Alverman A, Fehske H (2011). High-order commutator-free exponential time-propagation of driven quantum systems. J. Comput. Phys..

[2] Auzinger W, Březinová I, Hofstätter H, Koch O, Quell M (2019). Practical splitting methods for the adaptive integration of nonlinear evolution equations. Part II: comparisons of local error estimation and step-selection strategies for nonlinear Schrödinger and wave equations. Comput. Phys. Commun..

[3] Auzinger, W., Dubois, J., Held, K., Hofstätter, H., Jawecki, T., Kauch, A., Koch, O., Kropielnicka, K., Singh, P., Watzenböck, C.: Efficient Magnus-type integrators for Hubbard models of solar cells. Submitted

[4] Auzinger W, Herfort W (2014). Local error structures and order conditions in terms of Lie elements for exponential splitting schemes. Opusc. Math..

[5] Auzinger W, Hofstätter H, Ketcheson D, Koch O (2017). Practical splitting methods for the adaptive integration of nonlinear evolution equations. Part I: construction of optimized schemes and pairs of schemes. BIT.

[6] Auzinger, W., Hofstätter, H., Koch, O.: Coefficients of various splitting methods (2017). http://www.asc.tuwien.ac.at/~winfried/splitting/10.1007/s10543-016-0626-9 PMC6407747 30930704

[7] Auzinger W, Hofstätter H, Koch O, Quell M, Thalhammer M (2019). A posteriori error estimation for Magnus-type integrators. M2AN Math. Model. Numer. Anal..

[8] Auzinger W, Hofstätter H, Koch O, Thalhammer M (2014). Defect-based local error estimators for splitting methods, with application to Schrödinger equations, part III: the nonlinear case. J. Comput. Appl. Math..

[9] Auzinger W, Koch O, Quell M (2017). Adaptive high-order splitting methods for systems of nonlinear evolution equations with periodic boundary conditions. Numer. Algorithms.

[10] Auzinger W, Koch O, Thalhammer M (2012). Defect-based local error estimators for splitting methods, with application to Schrödinger equations, part I: the linear case. J. Comput. Appl. Math..

[11] Auzinger W, Koch O, Thalhammer M (2013). Defect-based local error estimators for splitting methods, with application to Schrödinger equations, part II: higher-order methods for linear problems. J. Comput. Appl. Math..

[12] Auzinger W, Koch O, Thalhammer M (2015). Defect-based local error estimators for high-order splitting methods involving three linear operators. Numer. Algorithms.

[13] Baleanu D, Ghanbari B, Assad J, Jajarmi A, Pirouz HM (2020). Planar system-masses in an equilateral triangle: numerical study within fractional calculus. CMES Comput. Model. Eng. Sci..

[14] Baleanu D, Jajarmi A, Sajjadi SS, Assad JH (2020). The fractional features of a harmonic oscillator with position-dependent mass. Commun. Theor. Phys..

[15] Berger T, Ilchmann A (2013). On the standard canonical form of time-varying linear DAEs. Quart. Appl. Math..

[16] Blanes S, Casas F (2006). Splitting methods for non-autonomous separable dynamical systems. J. Phys. A: Math. Gen..

[17] Blanes S, Casas F, Murua A (2007). Splitting methods for non-autonomous linear systems. Int. J. Comput. Math..

[18] Blanes S, Casas F, Murua A (2012). Splitting methods in the numerical integration of non-autonomous dynamical systems. Rev. R. Acad. Cien. Serie A Math..

[19] Blanes S, Casas F, Thalhammer M (2017). High-order commutator-free quasi-Magnus exponential integrators for nonautonomous linear evolution equations. Comput. Phys. Commun..

[20] Blanes S, Diele F, Marangi C, Ragni S (2010). Splitting and composition methods for explicit time dependence in separable dynamical systems. J. Comput. Appl. Math..

[21] Blanes S, Moan PC (2000). Splitting methods for the time-dependent Schrödinger equation. Phys. Lett. A.

[22] Blanes S, Moan PC (2005). Fourth- and sixth-order commutator-free Magnus integrators for linear and non-linear dynamical systems. Appl. Numer. Math..

[23] Hairer E, Lubich Ch, Wanner G (2006). Geometric Numerical Integration.

[24] Hairer E, Nørsett SP, Wanner G (1987). Solving Ordinary Differential Equations I.

[25] Held K (2007). Electronic structure calculations using dynamical mean field theory. Adv. Phys..

[26] Hofstätter H, Auzinger W, Koch O, England M, Koepf W, Sadykov TM, Seiler WM, Vorozhtsov EV (2019). An algorithm for computing coefficients of words in expressions involving exponentials and its application to the construction of exponential integrators. Computer Algebra in Scientific Computing, Lecture Notes in Computer Science.

[27] Innerberger, M.: Modeling of solar cells by small Hubbard clusters. B.Sc. Thesis, Vienna University of Technology (2017)

[28] Iserles, A., Kropielnicka, K., Singh, P.: On the discretisation of the semiclassical Schrödinger equation with time-dependent potential. http://www.damtp.cam.ac.uk/user/na/NA_papers/NA2015_02.pdf (2015)

[29] Jajarmi A, Baleanu D (2020). A new iterative method for the numerical solution of high-order nonlinear fractional boundary value problems. Front. Phys..

[30] Jawecki T, Auzinger W, Koch O (2020). Computable upper error bounds for Krylov approximations to matrix exponentials and associated $$\varphi $$-functions. BIT.

[31] Kauch, A., Worm, P., Prauhart, P., Innerberger, M., Watzenböck, C., Held, K.: Enhancement of impact ionization in Hubbard clusters by disorder and next-nearest-neighbor hopping. ArXiv preprint https://arxiv.org/abs/2007.16035

[32] Koch O, Neuhauser Ch, Thalhammer M (2013). Embedded split-step formulae for the time integration of nonlinear evolution equations. Appl. Numer. Math..

[33] Kyoseva E, Vitanova N, Shore B (2007). Physical realization of coupled Hilbert-space mirrors for quantum-state engineering. J. Modern Opt..

[34] Lakhel EH, McKibben MA (2019). Controllability for time-dependent neutral stochastic functional differential equations with Rosenblatt process and impulses. Int. J. Control Autom. Syst..

[35] Magnus W (1954). On the exponential solution of differential equations for a linear operator. Commun. Pure Appl. Math..

[36] Moler C, Van Loan CF (2003). Nineteen dubious ways to compute the exponential of a matrix, twenty-five years later. SIAM Rev..

[37] Press WH, Flannery BP, Teukolsky SA, Vetterling WT (1988). Numerical Recipes in C—The Art of Scientific Computing.

[38] Sajjadi SS, Baleanu D, Jajarmi A, Pirouz HM (2020). A new adaptive synchronization and hyperchaos control of a biological snap oscillator. Chaos Solitons Fractals.

[39] Seydaoglu M, Blanes S (2014). High-order splitting methods for separable non-autonomous parabolic equations. Appl. Numer. Math..

[40] Shapiro M, Brumer P (2003). Principles of the Quantum Control of Molecular Processes.

